# *Leptospira* Exposure and Gardeners: A Case-Control Seroprevalence Study

**DOI:** 10.14740/jocmr2377w

**Published:** 2015-12-03

**Authors:** Cosme Alvarado-Esquivel, Jesus Hernandez-Tinoco, Luis Francisco Sanchez-Anguiano, Agar Ramos-Nevarez, Sandra Margarita Cerrillo-Soto, Carlos Alberto Guido-Arreola

**Affiliations:** a Biomedical Research Laboratory, Faculty of Medicine and Nutrition, Juarez University of Durango State, Durango, Mexico; b Institute for Scientific Research “Dr. Roberto Rivera Damm”, Juarez University of Durango State, Durango, Mexico; c Clinica de Medicina Familiar, Instituto de Seguridad y Servicios Sociales de los Trabajadores del Estado, Predio Canoas S/N, 34079 Durango, Mexico

**Keywords:** *Leptospira*, Epidemiology, Seroprevalence, Gardeners, Case-control study, Mexico

## Abstract

**Background:**

*Leptospira* can be found in soil. However, it is unclear whether occupational exposure to soil may represent a risk for *Leptospira* infection in humans. Therefore, we sought to determine the association of *Leptospira* IgG seroprevalence with the occupation of gardener, and to determine the epidemiological characteristics of gardeners associated with *Leptospira* exposure.

**Methods:**

We performed a case-control study in 168 gardeners and 168 age- and gender-matched control subjects without gardening occupation in Durango City, Mexico. The seroprevalence of anti-*Leptospira* IgG antibodies in cases and controls was determined using an enzyme immunoassay. Bivariate and multivariate analyses were used to assess the association of *Leptospira* exposure and the characteristics of the gardeners.

**Results:**

Anti-*Leptospira* IgG antibodies were found in 10 (6%) of 168 gardeners and in 15 (8.9%) of 168 control subjects (odds ratio (OR): 0.64; 95% confidence interval (CI): 0.28 - 1.48; P = 0.40). Multivariate analysis showed that *Leptospira* seropositivity was positively associated with female gender (OR: 5.82; 95% CI: 1.11 - 30.46; P = 0.03), and negatively associated with eating while working (OR: 0.21; 95% CI: 0.05 - 0.87; P = 0.03). In addition, multivariate analysis showed that high anti-*Leptospira* levels were associated with consumption of boar meat (OR: 28.00; 95% CI: 1.20 - 648.80; P = 0.03).

**Conclusions:**

This is the first case-control study of *Leptospira* exposure in gardeners. Results do not support an association of *Leptospira* exposure with the occupation of gardener. However, further studies to confirm the lack of this association are needed. The potential role of consumption of boar meat in *Leptospira* infection deserves further investigation.

## Introduction

The pathogenic bacteria of the genus *Leptospira* cause morbidity and mortality worldwide [1]. Leptospirosis, the disease caused by *Leptospira*, is considered the most widespread zoonosis in the world [2]. The higher incidence of leptospirosis occurs in the tropics and subtropics [3]. Humans become infected with *Leptospira* by contact with *Leptospira*-infected animals or their urine [3, 4]. Evidence of *Leptospira* infection in virtually all mammalian species examined has been found [2]. Infection can also be acquired from the environment contaminated with *Leptospira*, i.e., soil and water [2, 5, 6]. Leptospirosis outbreaks have been associated with common water events including occupational exposure, water sports, consumption of water, and water disasters [7]. Infection with *Leptospira* occurs through skin abrasions [8]. The clinical spectrum of *Leptospira* infection varies from subclinical to life-threatening disease [9]. Clinical presentation of leptospirosis includes fever, flu-like symptoms, headache, intense myalgia, and liver involvement with jaundice and kidney involvement [8, 9].

*Leptospira* shed in the urine of infected animals may contaminate soil of gardens. These places are often visited by domestic and wild animals that may be infected with *Leptospira*, i.e., rats [1, 10, 11], dogs [12, 13], and cats [14]. Gardeners have a frequent contact with water and soil. Untreated water (sometimes from rivers or lakes) is used to water trees and plants in gardens in Durango, Mexico. Gardeners occasionally suffer from skin abrasions while working. Therefore, gardeners might have a risk for *Leptospira* infection. However, to the best of our knowledge, the association of *Leptospira* exposure with gardener occupation has not been studied. Therefore, this study aimed to determine the association of *Leptospira* IgG seropositivity and gardener occupation in Durango City, Mexico. The socio-demographic, work, clinical, and behavioral characteristics of the gardeners exposed to *Leptospira* were also investigated.

## Materials and Methods

### Study design and study populations

This serosurvey has an age- and gender-matched case-control study design. We used residual serum samples from *Toxoplasma gondii* serosurveys in gardeners [15] and general population [16] in Durango City, Mexico. The proportion of cases and controls was 1:1. Thus, cases included 168 gardeners, and controls included 168 subjects of the general population without gardener occupation. Participants were studied for the presence of anti-*Leptospira* IgG antibodies. Inclusion criteria for gardeners were: 1) subjects who have been working as gardeners for at least 6 months in Durango City, Mexico; 2) aged 16 years and older; 3) any gender; and 4) who accepted to participate in the study. Socioeconomic status, or work place (private or government) were not restrictive criteria for enrollment. As a strategy to enroll gardeners, they were visited at their work places in streets, boulevards, parks and work offices. Gardeners were 16- 73 (mean: 38.15 ± 13.91) years old, 15 were females and 153 were males. The control group included 168 subjects randomly selected from the general population of Durango City matched with cases by age and gender. Thus, the control group included 15 females and 153 males aged 38.10 ± 14.00 (range: 16 - 73) years old. No difference in age among cases and controls was found (P = 0.97).

### General characteristics of gardeners

Socio-demographic, work, clinical, and behavioral characteristics of gardeners were obtained from archival questionnaires [15]. Socioeconomic data included age, gender, birthplace, and educational level. In addition, the type of flooring at home was obtained. Work data included duration (years) in the activity, frequency of contact with water, sewage and soil, history of splashes at face with water, eating while working, washing hands before eating, history of injuries with sharp material at work, and use of safety practices (hand gloves, face masks and glasses). Clinical data included health status, history of blood transfusion or transplant history, and impairments in memory, reflexes, hearing and vision. Behavioral data included animal contacts, consumption of meat and type of meat consumed, degree of meat cooking, consumption of untreated water, unpasteurized milk or unwashed vegetables and fruits, eating away from home (in restaurants or fast food outlets), and traveling.

### Detection of *Leptospira* antibodies in serum

Serum samples of gardeners and controls were analyzed for detection of anti-*Leptospira* IgG antibodies using a commercially available enzyme-linked immunosorbent assay kit, “*Leptospira* IgG ELISA test” (Diagnostic Automation Inc., Calabasas, CA). According to the kit’s insert, this assay has a sensitivity of 100% and a specificity of 100%. An absorbance reading of ≥ 0.5 optical density (OD) units was used as a cut-off for positivity of anti-*Leptospira* IgG antibodies. High levels of anti-*Leptospira* IgG antibodies in sera were considered when absorbance readings > 1.0 OD units were obtained. All tests were performed according to the instructions of the manufacturer, and positive and negative controls were included in each run.

### Statistical analysis

Data were analyzed using the software Epi Info version 7 and SPSS version 15.0. Frequencies between groups were compared with the Fisher’s exact test. Age among groups was compared with the Student’s *t*-test. The association between the characteristics of gardeners and *Leptospira* exposure was assessed by bivariate and multivariate analyses. Variables with P values < 0.10 obtained in the bivariate analysis were selected for inclusion in the multivariate analysis. Logistic regression analysis with the Enter method was used to calculate the odds ratios (ORs) and 95% confidence intervals (CIs). A P value < 0.05 was considered statistically significant. Two multivariate analysis models were used: one to assess the association of *Leptospira* exposure with the gardeners’ characteristics, and other to assess the association of high anti-*Leptospira* antibody levels with the gardeners’ characteristics. The Hosmer-Lemeshow goodness of fit test was used to assess the fitness of our regression models.

### Ethical aspects

We studied only archival sera and data of participants from previous surveys [15, 16]. This study was approved by the Ethical Committee of the Instituto de Seguridad y Servicios Sociales de los Trabajadores del Estado in Durango City. We obtained a written informed consent from major participants and from the next of kin of minor participants.

## Results

Anti-*Leptospira* IgG antibodies were found in 10 (6%) of 168 gardeners and in 15 (8.9%) of 168 control subjects. No statistically significant difference in *Leptospira* seroprevalence among cases and controls was found (OR: 0.64; 95% CI: 0.28 - 1.48; P = 0.40). The frequency of high levels (≥ 1.0 OD units) of anti-*Leptospira* IgG antibodies in gardeners (3/168: 1.8%) was also similar to that (4/168: 2.4%) in control subjects (OR: 0.74; 95% CI: 0.16 - 3.38; P = 1.00). Stratification by gender showed that three (20%) of 15 female cases and null of 15 female controls were positive for anti-*Leptospira* antibodies (P = 0.22). On the other hand, seven (4.6%) of 153 male cases and 15 (9.8%) of 153 male controls were positive for anti-*Leptospira* antibodies (P = 0.11).

Of the socio-demographic characteristics of gardeners, bivariate analysis showed that *Leptospira* exposure was associated with gender. Female gardeners had a significantly higher seroprevalence of *Leptospira* infection than male gardeners did (20% vs. 4.6%, respectively: P = 0.04). Other socio-demographic characteristics of gardeners including age, birthplace, and education, and type of flooring at home showed P values ≥ 0.10 by bivariate analysis.

With respect to work characteristics of gardeners, two variables showed P values < 0.10 by bivariate analysis: eating while working (P = 0.03), and frequency of contact with water (P = 0.09). Other work characteristics of gardeners including duration in the activity, frequency of contact with sewage and soil, history of splashes at face with water, washing hands before eating, history of injuries with sharp material at work, and use of safety practices showed P values ≥ 0.10 by bivariate analysis.

None of the clinical characteristics including health status, history of blood transfusion or transplant history, and impairments in memory, reflexes, hearing and vision was associated with *Leptospira* exposure by bivariate analysis. Concerning behavioral characteristics of gardeners, the variable consumption of boar meat showed a P value of 0.06 by bivariate analysis. Whereas the variables including animal contacts, consumption of meat other than boar meat, degree of meat cooking, consumption of untreated water, unpasteurized milk or unwashed vegetables and fruits, eating away from home, and traveling showed P values ≥ 0.10 by bivariate analysis. Multivariate analysis of the four variables (gender, eating while working, frequency of contact with water, and consumption of boar meat) with P values < 0.10 obtained in the bivariate analysis showed that *Leptospira* seroreactivity was positively associated with female gender (OR: 5.82; 95% CI: 1.11 - 30.46; P = 0.03), and negatively associated with eating while working (OR: 0.21; 95% CI: 0.05 - 0.87; P = 0.03). The result of the Hosmer-Lemeshow test (P = 0.61) suggested a good fitting of this regression model.

Bivariate analysis of the association of the gardeners’ characteristics and high (> 1.0 OD units) anti-*Leptospira* IgG antibody levels showed three variables with P values < 0.10: consumption of boar meat (P = 0.02), national trips (P = 0.05), and consumption of sheep meat (P = 0.07). Multivariate analysis of these characteristics showed that high (> 1.0 OD units) anti-*Leptospira* IgG antibody levels were associated only with consumption of boar meat (OR: 28.00; 95% CI: 1.20 - 648.80; P = 0.03). The result of the Hosmer-Lemeshow test (P = 1.00) suggested a good fitting of this regression model.

## Discussion

The seroepidemiology of *Leptospira* infection in workers occupationally exposed to soil is largely unknown. We are not aware of any report of descriptive or case-control studies on the seroprevalence of *Leptospira* infection in gardeners. Therefore, this study aimed to determine whether gardeners are at a higher risk for *Leptospira* infection than subjects without this occupation are. We found that gardeners had a similar seroprevalence of *Leptospira* exposure to controls. This result was unexpected since gardeners have a number of known risk factors for *Leptospira* exposure including contact with soil [5, 8], and water [2, 7]. In addition, many gardeners studied did not use safety practices as wearing gloves, facemasks or glasses for avoiding infection. Skin abrasion is an important route of *Leptospira* infection [8]. However, injuries at work with sharp materials were not associated with *Leptospira* exposure in gardeners in our study. Furthermore, a considerable number of animals that may contaminate soil with their urine frequently visit gardens. The seroprevalence of *Leptospira* infection found in gardeners (6%) is also comparable with seroprevalences reported in waste pickers (4.4%) and their controls (5.6%) in the same Durango City [17]. In contrast, the seroprevalence found in gardeners is lower than the 15.6% seroprevalence reported in general population in rural Durango [18]. Therefore, results suggest that gardeners do not have an increased risk for *Leptospira* exposure. The lack of studies about the epidemiology of *Leptospira* infection in gardeners does not allow us to compare our results with others, and additional studies to confirm or challenge our results are needed. It is unclear why *Leptospira* seropositivity was not associated with gardener occupation. Many animals urinate in gardens, and the number of animals with this practice may be higher in public gardens than in house gardens. Many of the gardeners studied worked in parks where dogs and other animals are seen daily. Rodents are considered the most important reservoir of *Leptospira* infection [4, 8, 11]. Thus, it is possible that the number of rodents in work places of gardeners was low, or the frequency of *Leptospira* infection in these animals was null or too low. It is also possible that the frequency of *Leptospira* infection in animals other than rodents living in or visiting the gardens was quite low, and thus soil contamination with *Leptospira* through urine of animals was low too. We are not aware of any study of *Leptospira* infection in animals in Durango, and the prevalence of this infection in rodents, dogs and other animals in Durango is largely unknown.

Analysis of the association of *Leptospira* seropositivity and gardeners’ characteristics showed that *Leptospira* exposure was positively associated with female gender and negatively associated with eating while working. *Leptospira* seroprevalence has been associated with male gender [19, 20]. However, to the best of our knowledge, this is the first study that found an association of *Leptospira* seropositivity and female gender. It is unclear why female gardeners had a higher seroprevalence than male gardeners did. It is possible that female gardeners had different risk factors for *Leptospira* infection than male gardeners did. However, the number of females studied (n = 15) was low to perform a more extensive analysis of risk factors. Most gardeners in Durango City are males, and this was the reason we included more male gardeners than female gardeners in our study. On the other hand, the negative association of *Leptospira* exposure with eating while working suggests that this practice had no importance for *Leptospira* exposure among gardeners. Interestingly, multivariate analysis showed that high anti-*Leptospira* levels were associated with consumption of boar meat. We are not aware of any seroepidemiology study that had reported this association. However, infections with *Leptospira* have been demonstrated in boars. *Leptospira* DNA was found in kidneys of boars in Japan [21]. In addition, anti-*Leptospira* antibodies were found in 65.4% of wild boars in Portugal [22], and in 33% of feral hogs (*Sus scrofa*) in Florida, USA [23]. Results of the epidemiological link between consumption of boar meat and *Leptospira* exposure found in this study warrant for further research.

### Conclusions

This is the first case-control study of *Leptospira* exposure in gardeners. Results do not support an association of *Leptospira* exposure with the occupation of gardener. However, further research to confirm the lack of this association is needed. The potential role of the consumption of boar meat in *Leptospira* infection in humans deserves further investigation.
